# A multifactorial dimensionality reduction model for gene polymorphisms and environmental interaction analysis for the detection of susceptibility for type 2 diabetic and cardiovascular diseases

**DOI:** 10.1186/1755-8166-7-S1-P116

**Published:** 2014-01-21

**Authors:** Badaruddoza, Basanti Barna, Kawaljit Matharoo, Amarjeet Singh Bhanwer

**Affiliations:** 1 Department of Human Genetics; Guru Nanak Dev University; Amritsar-143005, Punjab, India

## Background

The present study has presented a comparative gene-environment interactions such as three genes (*ENPP1*-K121Q, *TCF7L2*-G>T and GYS1 A1>A2) and six environmental factors (obesity and cardiovascular related risk factors) for the detection of susceptibility for T2DM and CVD and for the interpretation of epistasis involved in genetic studies of disease susceptibility.

## Materials and methods

The final sample size included 250 cases and 250 controls to focus on better methodological quality and a higher statistical power analysis. All the interactions and approaches were carried out using the methods of MDR.

## Results

The MDR analysis showed the interaction between environmental factor (SBP) and the genetic factor (*ENPP1*) for pooled and female T2DM patients which indicated that SBP and *TCF7L2* had significant contribution on susceptibility to T2DM. The analysis showed that environmental factors (BMI, WHR, WC, SBP, DBP and PR) and genetic factors (*ENPP1*-K121Q, *TCF7L2* -G>T and GYS1 A1>A2) have identified risk factors and their interaction. All these interactions were observed to be significant. The MDR method showed all interaction models first to ninth order interactions for pooled and male T2DM patients as significant for susceptibility of obesity. Whereas in female T2DM patients, a first order (WHR) and third order (WHR * SBP * *ENPP1*) have found a significant interaction for obesity. Both the genes *ENPP1* and *TCF7L2* interacting with WHR and WC increase the susceptibility of obesity many folds among T2DM patients and non-diabetic controls. These results were also supported by dendrogram and interaction entropy model. The three factor interaction model (BMI * SBP * *ENPP1*) for pooled T2DM patients have been found significant for predicting hypertension in T2DM patients whereas, in female T2DM patients all the interaction models have been found as significant. However, third order model (SBP * *TCF7L2* * GYS1) have been found as a strong predictor for hypertension. In T2DM male patients there has been no significant interaction observed for gene-environment interaction although a seven factor model (BMI * WHR * WC * SBP * PR * ENPP1 * TCF7L2) seems to be comparatively a good predictor for hypertension.

## Conclusions

The results showed that both the genes *ENPP1* and *TCF7L2* interacting with WHR and WC increase the susceptibility of obesity many folds among T2DM patients and non-diabetic controls.

